# An approach to heart failure for the public-sector primary care clinician

**DOI:** 10.4102/safp.v67i1.6126

**Published:** 2025-06-13

**Authors:** Liezel Rossouw, Anthony S. Lachman, Klaus B. von Pressentin

**Affiliations:** 1 Division of Family Medicine and Primary Care, Stellenbosch University, Cape Town, South Africa; 2 Private Practice, Cardiology, Cape Town, South Africa; 3 Division of Family Medicine, Department of Family, Community and Emergency Care, University of Cape Town, Cape Town, South Africa

**Keywords:** heart failure, cardiac failure, primary care, public sector, primary care clinician, heart failure management, primary healthcare, clinical approach, heart failure guidelines, public health, primary care practice, district hospital, district level

## Abstract

Heart failure poses a significant global health challenge, with a considerable burden in Africa, where the annual mortality rate stands at 34%, twice the global average. Patients suffering from acute heart failure occupy numerous beds at the district level, and only a limited number can be referred for further evaluation and imaging at secondary or tertiary care facilities. Patients rely on their primary care physicians for the diagnosis and management of heart failure, as well as for identifying those who would benefit from referral to cardiology and formal echocardiography. This article discusses the significance of the new heart failure guidelines within the South African primary care public setting. It emphasises the importance of identifying risk factors and considers the value of access to family physicians, outreach clinic doctors, training on available adult primary care guidelines and telemedicine-supported cardiac ultrasound. Optimal medical therapy, which includes angiotensin-converting enzyme (ACE) inhibitors, beta-blockers and spironolactone, has been shown to reduce readmissions and mortality rates. Sodium-glucose cotransporter-2 (SGLT2) inhibitors are a potent addition to conventional therapy and are currently being considered for inclusion in the National Essential Medicines List. Patients admitted to the hospital should not be discharged while experiencing persistent congestion, as this is associated with an increased risk of rehospitalisation, mortality and higher healthcare costs. Comprehensive patient education regarding medications, thorough follow-up during the six weeks post-discharge and linkage to primary healthcare are associated with decreased hospitalisation rates and improved outcomes.

## Introduction

In 2018, cardiovascular disease was South Africa’s leading non-communicable cause of death, rising from 54 701 deaths in 1997 to 80 133 in 2018.^1^ Africa has the highest chronic heart failure fatality rate at 34%, double the global average of 16.5%.^2^ Mortality peaks in productive ages, with a five year mortality rate of 42% – 60%, worse than most cancers.^3^

A 2018 study at a tertiary hospital in Cape Town examined the causes of heart failure and patient mortality.^4^ The 180-day readmission rate was 25.2%, with a 6-month case fatality rate of 26.1%. Most patients were prescribed renin-angiotensin blockers and loop diuretics at discharge; however, the use of beta-blockers, aldosterone antagonists and digoxin was relatively low.^4^ Factors related to readmission at another Cape Town tertiary hospital revealed a 30 day readmission rate of 10.5%, with 66% admitted within 14 days and 35% having potentially avoidable factors.^5^ According to the American College of Cardiology Foundation, post-discharge readmission rates are 20% at three months and 30% at six months.^6^ Similarly, mortality rates are 10% at three months and 20% at six months. The increased frequency of acute events leads to high rates of hospitalisation and decreased function; therefore, early identification and treatment of acute heart failure are crucial.^5,6^

The readmission and mortality rates of heart failure patients in South African district hospitals are poorly understood. In a district hospital in the Eastern Cape, a retrospective record review revealed that the prevalence of inpatient cardiovascular disease diagnoses was 27%, with hypertensive heart disease and heart failure representing 33.4% of cardiovascular diagnoses.^7^ However, despite this burden on the district health services, access to appropriate specialist care for patients with cardiac failure remains inadequate.

Primary care physicians and clinical nurse practitioners often lack sufficient skills or experience and have limited access to point-of-care decision-making tools; such as tests, monitoring and imaging. Various factors make it challenging to follow recommended guidelines, including inadequate care coordination systems and access to specialists and outreach doctors.^8^ This leads to unnecessary readmissions, missed opportunities for diagnosing reversible pathologies, and increased morbidity and mortality. The impact is considerable, resulting in elevated costs for our healthcare system and patients.

Nationally, the Ideal Clinic programme focuses on infrastructure, drug availability and equipment, but does not prioritise quality of care. The ideal hospital programme in 2023/2024 reported that only 62 out of 391 (16%) facilities were considered ‘ideal’. Resource availability in provinces differs considerably. Some clinics have speciality-trained nurses who can follow the Adult Primary Care guidelines, including those for heart failure. Many clinics in rural provinces lack speciality-trained nurses in primary care or outreach doctors, and many district health facilities do not have a family physician.

This article examines the significance of the latest heart failure guidelines in South African primary care within the public sector. It aims to contribute to initiatives designed to enhance the capabilities of primary care physicians, enabling them to deliver evidence-based care in collaboration with primary care teams and specialist providers.

## Setting the scene with a district hospital experience

This patient scenario will help contextualise the discussion on managing heart failure from the district health hospital perspective. A 52-year-old female patient with a history of hypertension, chronic heart failure and atrial fibrillation has been attending a local rural clinic for at least 10 years. Her medication regimen included furosemide 40 mg twice daily, warfarin as per INR and enalapril 10 mg orally twice daily. Her chronic prescriptions were recently renewed without consulting a medical officer. In 2023, she visited the local district hospital’s emergency unit because of leg swelling and shortness of breath, with no other significant medical history noticed. During this acute episode, she presented with a blood pressure of 150/100 mmHg but lacked other apparent precipitating factors. Clinical evaluations confirmed biventricular heart failure. She was treated with intravenous furosemide 40 mg every 12 h, along with enalapril 10 mg daily and amlodipine 5 mg daily. Recommended blood tests returned unremarkable results, while a chest X-ray indicated pulmonary oedema, and the ECG showed atrial fibrillation at a rate of 120 beats per minute. Although she responded positively to intravenous diuresis, peripheral oedema persisted. Unfortunately, she was discharged from the hospital because of the pressure on inpatient beds.

One month later, she returned with similar symptoms, was unable to resume work and required re-admission. Her medication did not include amlodipine, as her previous chronic prescriptions had not been updated. In addition to her still uncontrolled blood pressure of 150/110 mmHg and pulse of 130 beats per minute, no other precipitating factors were identified. Her medications consisted of intravenous furosemide 80 mg twice daily, enalapril 20 mg daily, warfarin and amlodipine 5 mg orally daily. The family physician evaluated her on day three of admission, diagnosing heart failure with reduced ejection fraction because of uncontrolled hypertension and rapid atrial fibrillation. An informal bedside cardiac ultrasound was conducted via telemedicine with a referral hospital, confirming a severely dilated left ventricle with a significantly reduced ejection fraction but no apparent valve issues. It was determined that she would only receive maximal medical treatment without escalation of care. Spironolactone 25 mg orally and carvedilol were initiated at 3.125 mg po every 12 h once her crepitations subsided. However, because of bed pressures, a junior doctor discharged her before achieving full diuresis. The discharge medications included carvedilol 6.25 mg every 12 h, enalapril 20 mg daily, furosemide 80 mg every 12 h, spironolactone 25 mg orally daily, warfarin and amlodipine 10 mg daily.

Two months later, she presented to the hospital again with similar complaints. During this admission, the family physician issued clear instructions not to discharge the patient until full diuresis was achieved and to titrate the carvedilol in the ward to 12.5 mg orally every 12 h. Adequate diuresis was accomplished by monitoring weight and output. The patient was educated about her condition and the medications she was prescribed. She was closely monitored by the same family physician for six weeks, achieving optimal dosages for all her medications. Her medication regimen included enalapril 20 mg orally daily, carvedilol 25 mg orally every 12 h, amlodipine 10 mg orally daily, spironolactone 25 mg orally daily and furosemide 60 mg orally every 12 h. Her blood pressure on this medication was 110/70 mmHg, and her pulse was 60 beats per minute. Although she was scheduled for an echocardiogram in September 2024, it has been postponed to March 2025. Clear communication with the local clinic for treatment adherence was prioritised. As of now, she has had no acute admissions in 2024 or 2025 and has returned to work successfully.

## Understanding clinical heart failure patterns

A sound clinical understanding of preload and afterload is essential in identifying the correct management strategies when considering this patient scenario.^9^ Reviewing the classification of heart failure enhances this understanding. Moreover, this patient scenario highlights the importance of early identification, consultation with a doctor at the clinic level and appropriate treatment, particularly as the increasing frequency of acute events leads to high hospitalisation rates and decreased function.^10,11,12^

**FIGURE 1:**
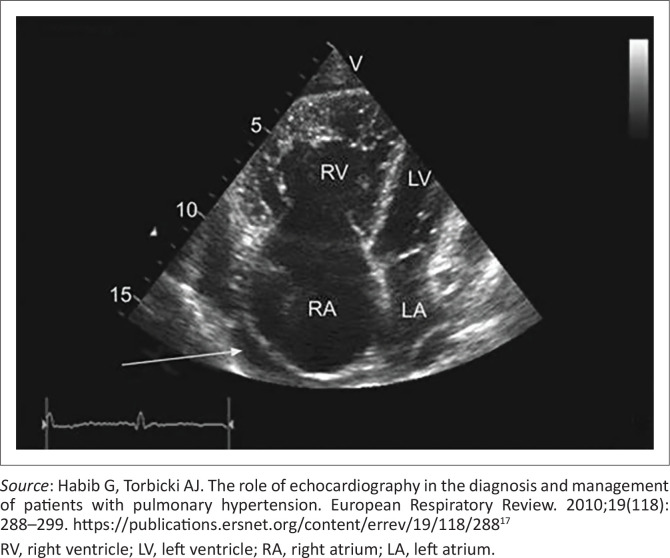
Right-sided heart failure, showing that the right ventricle appears larger than the left ventricle. In a normal heart, the right side is smaller in diameter than the left side.

**FIGURE 2:**
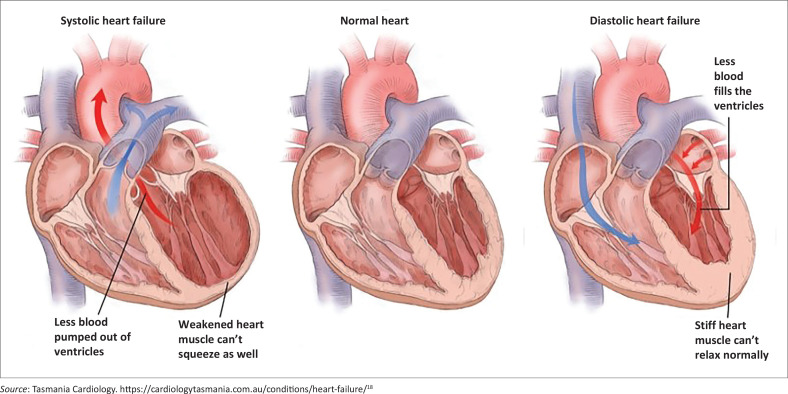
Systolic and diastolic cardiac failure.

Three commonly used methods for classifying heart failure are assessing symptoms and physical activity, clinically distinguishing between left- and right-sided heart failure, and evaluating ejection fraction using an echocardiogram. From a practical approach to treatment, distinguishing three clinical patterns may guide the clinician:

1. Right-sided heart failure (Figure 1).
2. Diastolic heart failure in the hypertensive patient presenting with dyspnoea and a normal or preserved ejection fraction and diastolic dysfunction (Heart Failure with preserved ejection fracture; HFpEF) (Figure 2).
3. Systolic heart failure with a reduced ejection fraction (Heart Failure with reduced ejection fracture; HFrEF) (Figure 2).

The severity of symptoms and physical activity is guided by the New York Heart Association (NYHA) functional classification, and the ejection fraction is based on the European Society of Cardiology (ESC) 2022, the American Heart Association (AHA) classification and the Heart Failure Society of South Africa (HeFFSA) (Table 1).^13,14,15,16^

**TABLE 1:** Definition of heart failure according to the European Society of Cardiology guidelines 2022.

Type of HF† and criteria	HFrEF†	HFmrEF†	HFpEF†
1	Symptoms ± Signs	Symptoms ± Signs	Symptoms ± Signs
2	LVEF ≤ 40%†	LVEF 41% – 49%†	LVEF ≥ 50%†
3	-	-	Objective evidence of cardiac structural and/or functional abnormalities consistent with presence of LV diastolic dysfunction/raised LV filling pressures, including raised natriuretic peptides

*Source*: Adapted from McDonagh TA, Metra M, Adamo M, et al. 2021 ESC guidelines for the diagnosis and treatment of acute and chronic heart failure. Eur J Heart Fail. 2022;24(1):4–131. https://doi.org/10.1002/ejhf.2333

† , heart failure; HFmrEF, heart failure with mildly reduced ejection fraction; HFrEF, heart failure reduced ejection fraction; LV, left ventricle; LVEF, left ventricular ejection fraction; HFpEF, heart failure with preserved ejection fracture.

## Diagnosing and investigating heart failure

A patient is suspected to have heart failure based on risk factors (Table 2), symptoms and an abnormal ECG. The history and clinical examination are crucial in identifying the precipitating factors and the underlying cause of cardiac failure (Table 3). However, leg swelling and dyspnoea can be caused by other conditions. A raised JVP, bilateral crepitations and an s3 (third heart sound) are more specific.^10^ The ECG is always abnormal in heart failure with a reduced ejection fraction.^13^ A CXR shows cardiomegaly and can exclude other causes of dyspnoea. Routine blood tests with Class 1 recommendations include a full blood count, renal function and electrolytes, thyroid function, fasting glucose, cholesterol and iron status.^13^ Important precipitants of heart failure and comorbidities to recognise include thyroid pathology, anaemia, renal failure, electrolyte dysfunction and uncontrolled blood pressure.^10^ Important precipitating causes to exclude (Table 3) are those that are treatable or can benefit from surgery, including coronary heart disease, valvular heart disease, constrictive pericarditis, sarcoidosis or amyloidosis.^10^

**TABLE 2:** Risk factors for the development of heart failure and potential corrective actions.

Risk factors for heart failure	Preventative strategies
Sedentary habit	Regular physical activity
Cigarette smoking	Cigarette smoking cessation
Obesity	Physical activity and healthy diet
Excessive alcohol intake	General population: no or light alcohol intake is beneficial Patients with alcohol-induced CMP should abstain from alcohol
Influenza	Influenza vaccination
Microbes (e.g. *Trypanosoma cruzi, Streptococci*)	Early diagnosis and specific antimicrobial therapy and preventative therapy
Cardiotoxic drugs	Cardiac function and side effect monitoring, dose adaptation, change of chemotherapy
Chest radiation	Cardiac function and side effect monitoring and dose adaptation
Hypertension	Lifestyle changes, antihypertensive therapy
Dyslipidaemia	Healthy diet, statins
Diabetes mellitus	Physical activity and healthy diet, SGLT2 inhibitors
CAD	Lifestyle changes and statin therapy

*Source*: Adapted from McDonagh TA, Metra M, Adamo M, et al. 2021 ESC Guidelines for the diagnosis and treatment of acute and chronic heart failure. Eur J Heart Fail. 2022;24(1):4–131. https://doi.org/10.1002/ejhf.2333

CAD, coronary artery disease; CMP, cardiomyopathy; SGLT2, sodium-glucose co-transporter 2.

**TABLE 3:** Precipitating common causes in primary care.

Cause	Example of presentation	Specific actions
Cardiomyopathy	Hypertrophic Dilated in peripartum or because of toxins, alcohol, cocaine, iron, copper	Peripartum cardiac failure needs urgent referral and discussion for bromocriptine. Hypertrophic or dilated cardiomyopathies start omt† and refer.
Ischaemia	Myocardial infarction or angina Ischaemic heart disease Arrythmias	Acute onset of heart failure following an ischaemic event needs to be referred more urgently. If ongoing symptoms of angina and heart failure on omt† refer for formal echo ± revascularisation.
Valvular	Congenital or rheumatic heart disease or valvular degeneration/stenosis	If acute heart failure, refer for formal echo as an inpatient, especially if heart failure is not settling. The identification of symptomatic tight aorta stenosis is important as it is easily corrected. A new non-invasive procedure, namely transcatheter aortic valve implantation (TAVI) is non-inferior to open heart surgery in certain patients for aortic stenosis.
Cor pulmonale	Right-sided heart failure	Formal sleep study needed before oxygen or ventilation strategies decided (contraindicated in central apnoea). Treat preload.
Hypertension	Treat with ACE-inhibitor	Treatment of hypertension is key.
Pericardial	Right-sided heart failure with pericardial effusion Constrictive pericarditis	Treatment directed at cause, refer for echo if cause unclear.
Arrhythmias	Can be atrial or ventricular Fast atrial fibrillation and cardiac failure is common	For atrial fibrillation, treat the triggers, anticoagulate and optimise heart failure treatment. Anticoagulation for all patients with AF and heart failure if the CHA2DS2-VASc scoreΩ is equal to or more than two in males or equal to or more than three in females. Rivaroxaban is preferred over warfarin except in mechanical valves and mitral stenosis patients. If haemodynamically unstable, electric cardioversion (ECV) is a Class I recommendation. If not unstable, rate control (HR < 110) with beta-blockers or digoxin or amiodarone. If no symptom improvement ablation or amiodarone or ECV is suggested. Acute onset atrial fibrillation can be referred for elective cardioversion.
Metabolic	Thyroid Thiamine, Vitamin B1 and selenium deficiencies Auto-immune	Treat the underlying cause in discussion with physicians. Thiamine is given.
Infective	Viral myocarditis HIV Lymes and Chagas	Acute myocarditis: Clinical presentation of chest pain, dyspnoea, signs of right or left heart failure and/or unexplained arrhythmias plus a mandatory diagnostic test including a Troponin T or ECG with new ST-T changes or arrhythmias, QRS abnormalities or blocks. Diagnosis and management are to be discussed with a physician and 48 h hospital observations. HIV is a cause of exclusion.
Infiltrative	Amyloidosis	Screen with serum protein, calcium and free light chains

*Source*: Adapted from McDonagh TA, Metra M, Adamo M, et al. 2021 ESC Guidelines for the diagnosis and treatment of acute and chronic heart failure. Eur J Heart Fail. 2022;24(1):4–131. https://doi.org/10.1002/ejhf.2333

† , omt, optimal medical therapy.

ΩCHA2DS2-VASc, A score for congestive heart failure, hypertension, age, diabetes, stroke, vascular disease, age and sex category to predict risk for stroke in people with atrial fibrillation; HIV, human immunodeficiency virus; ECG, electrocardiogram; ECV, electrical cardioversion; ACE, angiotensin-converting enzyme.

An NT-proBNP (N-terminal pro-Brain Natriuretic Peptide) is recommended in international guidelines for patients with suspected cardiac failure.^13,14,15,16^ In conjunction with signs, symptoms and ECG, the diagnostic value of NT-proBNPs has been assessed in primary care settings. An NT-proBNP of less than 125 pg/mL has a negative predictive value of 94% – 98%.^19,20^ Although elevated levels support the diagnosis, other causes may increase levels, including atrial fibrillation, increased age and renal failure. An NT-proBNP may be lower than expected in obese patients.^13^ An NT-proBNP of more or equal to 125 ng/mL is found in young females with no heart failure risk factors and should be interpreted with caution.^13^

If heart failure is suspected, but an NT-proBNP is unavailable, echocardiography is indicated to determine the aetiology of heart failure.^13^ The skill of echocardiography is not widely available, especially in rural areas. However, training in cardiac ultrasound enhances diagnosis. Utilising current technology through video sharing with a specialist cardiologist enhances the accuracy and interpretability of the data.^8^ Family physicians are well-positioned to ensure point-of-care ultrasound (POCUS) training and effective use while minimising possible harm.^19^ However, the limited number of family physicians employed is a significant obstacle to implementation.

The estimated average daily cost of a district hospital bed in South Africa is between R1219 and R1543 in 2024.^22^ An NT-proBNP test currently costs R569.02 in the public sector through the National Health Laboratory Services (NHLS). Increased admission times related to diagnostic work-up and potential transfers for imaging are common in patients admitted with shortness of breath. This highlights the possible value of utilising the NT-proBNP in district settings where cardiac failure can be excluded. Consideration should be given to the laboratory turnaround time for specific district hospitals, which varies considerably.

The use of NT-pro BNP is currently not included in the Essential Medication List and evidence of its use at the primary care level is lacking.

## Heart failure management

### General principles

The three essential steps in managing a patient with heart failure include an accurate and timely diagnosis of the disease, rapid treatment of patients in the acute setting and a focus on transitioning care, patient education and support.^6^

The management of heart failure will be addressed based on the clinical presentation classification:^13,14,15,23^

**FIGURE 3:**
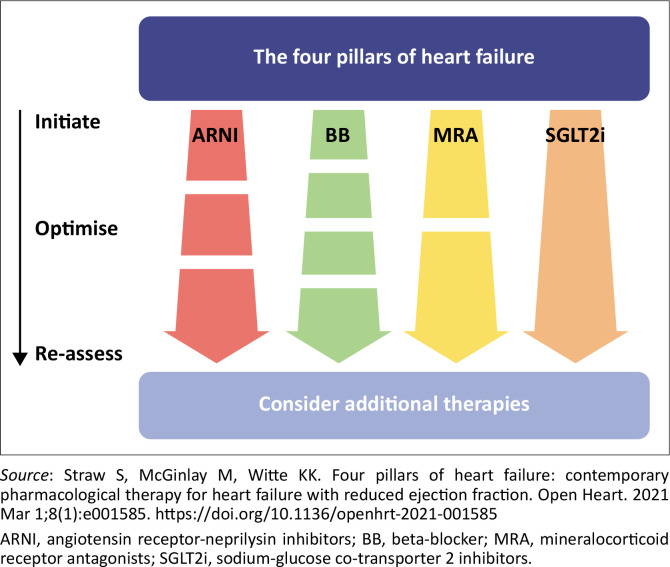
Initiation and optimisation of the four pillars of heart failure.

1. Right-sided heart failure (Figure 1).
  As it is mainly a preload problem, the preload is treated with furosemide. Spironolactone (mineralocorticoid-receptor antagonist [MRA]) can be added.
2. The hypertensive patient with dyspnoea and diastolic dysfunction with a normal ejection fraction (HFpEF) (Figure 2).
  The issue is the stiff and thick ventricle that cannot fill adequately. These patients clinically present with symptoms of cardiac failure, but the clinical diagnosis remains uncertain. The NT-proBNP levels are greater than or equal to 125 pg/mL; an echocardiogram can confirm the presence of diastolic dysfunction.
  - Screening for and managing aetiologies, along with cardiac and non-cardiac comorbidities (Table 2).^13^
  - Diuretics for patients with congestive heart failure to relieve signs and symptoms.^13^
3. Heart failure with a reduced ejection fraction (HFrEF) (Figure 2).
  The ventricle is stretched and thinned, preventing it from pumping efficiently. This results in a reduced ejection fraction; these patients exhibit clinical symptoms and signs of cardiac failure, and the diagnosis is beyond doubt.
  - Screen for and treat aetiologies and cardiac and non-cardiac comorbidities (Table 2).
  - Optimal Medical Therapy (the ‘Fabulous Four’ drugs or quadruple therapy described further in the text and in Figure 3) decreases hospitalisation and death^13,14,15^ Optimal Medical therapy is defined as the ability to add all four drugs and slowly titrating it to the highest recommended doses without the patient becoming symptomatic from a systolic blood pressure of less or equal to 90 and or a pulse of less than 60.
    ■ Angiotensin-converting enzyme (ACE) or angiotensin receptor blockers (ARB) or angiotensin receptor neprilysin inhibitor (ARNi).
  Angiotensin receptor neprilysin inhibitor is more effective than ACE inhibitors, and ACE inhibitors are more effective than ARBs in reducing hospitalisation and mortality. All four pillar drugs are more effective at low doses than fewer than four drugs at higher doses. Consider whether you are not beginning the four pillar drugs, and if so, why not? Can I titrate?
  - Beta-blockers (BBs) are initiated at a low dose in stable patients with no bibasal crepitations and gradually titrated to target as soon as possible. Historically, two standard classes of BBs are:
    ■ B1 receptor inhibitors (cardio-selective). Examples are atenolol, metoprolol succinate and bisoprolol.
    ■ B1 and B2 receptor inhibitors (non-cardio-selective). Examples are carvedilol, propranolol, labetalol, timolol.
    ■ In patients with cardiovascular disease and chronic obstructive airway disease (COAD), BBs are often under-prescribed because of concerns about respiratory side effects. Currently, carvedilol and bisoprolol are recommended in these patients.^24,25^ Patients with heart failure and asthma tolerate carvedilol poorly.^26,27^ The use of atenolol for heart failure patients with coronary artery disease and asthma should be discussed with a specialist.
  - A mineralocorticoid-receptor antagonist, namely spironolactone or eplerenone.
  - Caution is advised for patients with renal function (eGFR less than or equal to 30) and potassium levels exceeding 5 mmol/L.
  - Sodium-glucose cotransporter (SGLT2) inhibitors (dapagliflozin or empagliflozin).
  - Sodium-glucose co-transporter 2 inhibitors have been shown to decrease all-cause mortality by 25% and enhance physical function and quality of life.^13,14,15^ Sodium-glucose cotransporter 2 inhibitors alleviate congestion and may help reduce loop diuretic requirements. This drug is currently unavailable in the primary public sector. However, it is accessible at select public tertiary hospitals through special funding schemes. The use of SGLT2 inhibitors in public care is currently being reviewed by the National Essential Medicines List Committee (NEMLC).
  - Loop diuretics.
  - These diuretics are indicated in congested patients to relieve symptoms but do not affect morbidity and mortality.
  - Other drugs to consider in specific patients with heart failure in public care:
    ■ Digoxin may be considered for patients with symptomatic HFrEF in sinus rhythm despite ongoing treatment with the ‘fabulous four’. Levels should be monitored, and it is not recommended for elderly patients, those who are hypokalaemic, or individuals with a lean body weight mass.
    ■ Hydralazine may be used in patients with NYHA class III–IV who are already on an ACE/ARB, beta-blocker, and MRA to reduce the risk of hospitalisation and mortality.

#### Additional therapeutical considerations

Thiamine 100 mg is administered daily in heart failure patients with a history of excessive alcohol use.^13,14,15^

Screening for and treating anaemia and iron deficiency in heart failure patients is classified as a Class 1 recommendation.^13,14,15^

### Managing acute heart failure

The ESC algorithm for managing acute heart failure patients highlights the need to recognise cardiogenic shock and respiratory failure requiring inotropic or ventilatory support. It includes a mnemonic (CHAMPIT) to identify the cause of acute heart failure (see Box 1). In the case study provided, heart rate control is highlighted as the tachycardia worsens the ejection fracture and filling time.

BOX 1 Acute heart failure causes.

**CHAMPIT**

**C** Acute **C**oronary Syndrome

**H** Hypertensive emergency

**A** Arrhythmia

**M** Mechanical cause

**P** Pulmonary embolism

**I** Infections

**T** Tamponade

*Source*: Adapted from McDonagh TA, Metra M, Adamo M, et al. 2021 ESC guidelines for the diagnosis and treatment of acute and chronic heart failure. Eur J Heart Fail. 2022;24(1):4–131. https://doi.org/10.1002/ejhf.2333

Intravenous furosemide can be initiated at one to two times the oral dose.^13^ The dose can be doubled if the urine output is inadequate (100 mL/h–150 mL/h). The maximum daily dosage is 400 mg – 600 mg if there is no renal impairment or 1000 mg daily in cases of renal impairment. Thiazides can be added in patients with a poor response. Importantly, evidence suggests that patients with persistent congestion should not be discharged from the hospital, as this is associated with increased rehospitalisation rates and mortality.^13,28^ In our resource-constrained public health system, the ‘bed crisis’ discharge of these patients does not benefit the system or our patients.

### Non-pharmacological management strategies

Patient self-care education includes regular exercise, weight loss of 10% – 20% and optimising sleep health. A practical guide for monitoring the diuresis response is to weigh the patient twice a week, especially when adjusting diuretic dosages. Avoiding large volumes of fluid intake (1.5 L/day – 2 L/day) and preventing dehydration when unwell is essential. Patients should maintain a healthy diet, restrict salt intake to less than 5 g per day, avoid or minimise excessive alcohol consumption and ensure they are immunised against influenza and pneumococcal disease. Additionally, it is vital to cease smoking, tackle psychological challenges and empower patients to self-monitor early symptoms of heart failure.^13,29^

## Care coordination in primary care

### Managing multimorbidity within the multi-professional team and home environment

The management of this patient is complex and often necessitates remote specialist advice and coordination among various care providers. A multidisciplinary team approach is vital, informed by comprehensive primary health care principles, including palliative care when appropriate.

Equity of care should be advocated for regardless of where the patient presents. Integrating care between the clinic, hospital, community and emergency unit will enhance patient outcomes and ensure greater efficiency and cost-effectiveness.^30^

Closer relationships and patient case reviews between levels of care are essential as part of clinical governance practices. Senior clinicians should review protocols, develop training materials and conduct local audits.^6,30,31^

Educating patients and caregivers to ensure treatment adherence and early identification of symptoms could be achieved.

### Knowing when to refer and ensuring sound follow-up arrangements

Generally, referrals should be organised if specialised treatment and diagnostic work-up are required to identify treatable and reversible causes.^23^ This includes:

- All patients with cardiac murmurs should be assessed, as this could be a reversible cause of heart failure.^13,23^
- Patients where an ischaemic aetiology for heart failure is suspected.^13,23^
- Patients with left bundle branch block on ECG and heart failure are potential candidates for cardiac resynchronisation therapy.^13,23^
- If peripartum cardiomyopathy is suspected, prompt consultation for a formal echocardiogram and initiation of bromocriptine for breastfeeding cessation is advised.^13,14,15^

The STRONG HF trial highlights the need for close follow-up of patients during the first 6 weeks after discharge to up-titrate oral therapy, as this reduces readmission and all-cause mortality.^32^

## Reviewing the typical district hospital patient scenario

Heart failure patients should be regarded as high-risk individuals, and systems should be implemented to prevent prescribing without consultation. Regular family physician ward rounds, where available, facilitate the review of heart failure patients and the optimisation of therapy. Good communication with the patient and their local clinic regarding their change in medication and adherence is essential. Local ongoing training regarding guidelines will enhance patient and efficiency outcomes. It should be emphasised that these patients are not suitable candidates for bed crisis discharges. Close follow-up by a senior or family physician for 6 weeks is essential.

## Recommendations

The most crucial strategy is to focus on preventing heart failure. Risk factors for heart failure, especially hypertension and alcohol abuse, should be identified and actively managed at the primary care level.

Identifying heart failure symptoms and signs, in conjunction with ECG findings, remains central to primary care management. The availability of functional ECG machines on the primary care platform through the ideal hospital and clinic programme should be addressed. Screening for and treating cardiac and non-cardiac comorbidities is essential for successful treatment.

Cardiac ultrasound skills and telemedicine advice from the immediate referral hospital will expedite definitive care and reduce unnecessary referrals, thus lowering costs associated with transfers and higher level care beds.

Quadruple therapy (ARNi or ACE-I or ARB, BB, MRA and SGLT2i) saves lives. All four drugs should be initiated and titrated to target doses in patients with heart failure and a reduced ejection fraction unless there is a clear contraindication. Close follow-up and communication with the local clinic will enhance treatment adherence.

Family physician posts need to be created at district hospitals and busy community healthcare centres in all provinces. Primary healthcare nurses should be supported by regular visits from doctors to ensure that patients with non-communicable diseases, such as heart failure, are adequately managed on the platform. Clinics should utilise available local guidelines, including the Practical Approach to Care Kit (PACK) and the Adult Primary Care Clinical Tool (APC), to enhance the quality of care. Ideal Clinic and hospital programmes should include aspects of quality of care.

## Conclusion

Access to ECG machines, cardiac ultrasound, family physicians and doctor outreach to the clinic, as well as training in available guidelines in primary care, impacts the management plan and care coordination for patients presenting with heart failure. Consequently, more patients can be managed and followed up appropriately and cost-effectively at the primary care level.
